# Supplementary material for: Dynamic transcriptomic profiles of zebrafish gills in response to zinc supplementation
Source: BMC Genomics. 2010 Oct 11;11:553. doi: 10.1186/1471-2164-11-553 (PMC3091702; doi:10.1186/1471-2164-11-553)
Supplement: Additional file 2 — Interactive Direct Interaction Network representing the molecular interactions between zinc, copper, iron, calcium and proteins encoded by transcripts changed by zinc supplementation. Mini web-site containing index.html and hyperlinked pages in subdirectory describing a Direct Interaction Network automatically generated based on curated interactions contained within the proprietary PathwayArchitect database. Ovals represent proteins and the circles symbolize metal ions. Objects are coloured by their abundance in zebrafish at the time-point they were significantly different from the control is a scale from -4 fold (dark green) to +4 fold (dark red). Where significant differences were found at more than one time-point, the colour overlay shows expression at the first instance. Dark blue squares denote 'binding', and light blue squares 'expression'; green squares stand for 'regulation', green diamonds for 'metabolism', and green circles for 'promoter binding'. Arrow heads indicate directionality of the interaction where annotated. All nodes and edges can be further interrogated by selecting the relative area of the image. [file 1471-2164-11-553-S2.zip › PathwayArchitect Zn xs DIN/160180.html]

# PROTEIN: Abcb1a

|  |  |
| --- | --- |
| Name | Abcb1a |
| Type | PROTEIN |
| Description | ATP-binding cassette, sub-family B (MDR/TAP), member 1A |
| Note | The membrane-associated protein encoded by this gene is a member of the superfamily of ATP-binding cassette (ABC) transporters. ABC proteins transport various molecules across extra- and intra-cellular membranes. ABC genes are divided into seven distinct subfamilies (ABC1, MDR/TAP, MRP, ALD, OABP, GCN20, White). This protein is a member of the MDR/TAP subfamily. Members of the MDR/TAP subfamily are involved in multidrug resistance as well as antigen presentation. This gene encodes a p-glycoprotein which actively transports a variety of hydrophobic amphipathic drugs and plays a major role in the blood-brain barrier permeability of certain drugs. |
| Alias | P-glycoprotein |
|  | multidrug resistance protein 1a |
|  | MDR3 |
|  | multi-drug resistance 3 |
|  | Pgp |
|  | Mdr1a |
|  | ATP-binding cassette, sub-family B (MDR/TAP), member 4 |
|  | Abcb4 |
|  | Pgy-3 |
|  | mdr-3 |
|  | P-gp |
|  | P glycoprotein 3 |
|  | multiple drug resistant 1a |
|  | Pgy3 |


---

|  |  |
| --- | --- |
| GO Component | integral to membrane |
|  | intercellular canaliculus |


---

|  |  |
| --- | --- |
| GO ID | GO:0006810 |
|  | GO:0005524 |
|  | GO:0042626 |
|  | GO:0046581 |
|  | GO:0042623 |
|  | GO:0015893 |
|  | GO:0000166 |
|  | GO:0017111 |
|  | GO:0016021 |
|  | GO:0016887 |


---

|  |  |
| --- | --- |
| Connectivity | 442 |


---

|  |  |
| --- | --- |
| Entrez ID | 170913 |
|  | 18671 |


---

|  |  |
| --- | --- |
| Agilent ID | A\_53\_P123944 |
|  | A\_53\_P161876 |
|  | A\_44\_P279083 |
|  | A\_53\_P145143 |
|  | A\_53\_P105085 |
|  | A\_51\_P155445 |
|  | A\_42\_P668953 |


---

|  |  |
| --- | --- |
| Cellular Localization | Membrane |
|  | Plasma membrane |
|  | Cell |


---

|  |  |
| --- | --- |
| Pathway | HIV |
|  | Zn xs inventory |
|  | Zn xs DIN |


---

|  |  |
| --- | --- |
| GO Process | transport |
|  | drug transport |


---

|  |  |
| --- | --- |
| DbXref | KEGG pathway##02010##ABC transporters - General##http://www.genome.jp/dbget-bin/show\_pathway?mmu02010+18671 |


---

|  |  |
| --- | --- |
| UniGene | Mm.207354 |
|  | Rn.82691 |


---

|  |  |
| --- | --- |
| Affymetrix Probeset ID | 102910\_at |
|  | 105261\_at |
|  | 1370464\_at |
|  | 1370465\_at |
|  | 1370583\_s\_at |
|  | 1419758\_at |
|  | 1419759\_at |
|  | m33581\_s\_at |
|  | S66618\_at |


---

|  |  |
| --- | --- |
| GO Function | ATPase activity, coupled to transmembrane movement of substances |
|  | ATPase activity, coupled |
|  | nucleoside-triphosphatase activity |
|  | ATPase activity |
|  | nucleotide binding |
|  | ATP binding |


---

|  |  |
| --- | --- |
| Nucleotide | NM\_133401 |
|  | M24417 |
|  | AK008292 |
|  | M33580 |
|  | X61103 |
|  | AY864315 |
|  | AY582535 |
|  | M33581 |
|  | S66618 |
|  | AK144907 |
|  | NM\_011076 |
|  | AF257746 |
|  | M30697 |
|  | AF286167 |


---

|  |  |
| --- | --- |
| Protein | AAS91649 |
|  | AAA39514 |
|  | NP\_596892 |
|  | BAE26128 |
|  | AAF69007 |
|  | NP\_035206 |
|  | BAB25582 |
|  | AAA39518 |
|  | AAA39517 |
|  | CAA43415 |
|  | AAK83023 |
|  | P21447 |
|  | AAW56448 |
|  | AAA03243 |


---

|  |  |
| --- | --- |
| Organism | Mammal |


---

|  |  |
| --- | --- |
| Location | chromosome 5, 5 1.0 cM, 5 A1 (Mus musculus) |
|  | 5 1.0 cM (Mus musculus) |
|  | chromosome 4, 4q12 (Rattus norvegicus) |


---

|  |  |
| --- | --- |
